# Presenteeism in Non-Academic Staff in a Public University Context: Prevalence, Associated Factors, and Reasons to Work While Sick during the COVID-19 Pandemic

**DOI:** 10.3390/ijerph192214966

**Published:** 2022-11-14

**Authors:** Sónia Magalhães, Joselina Barbosa, Elisabete Borges

**Affiliations:** 1 ICBAS—School of Medicine and Biomedical Sciences, University of Porto, 4050-313 Porto, Portugal; 2 Faculty of Medicine, University of Porto, 4200-319 Porto, Portugal; 3 Nursing School of Porto, ESEP, 4200-072 Porto, Portugal; 4 Center for Health Technology and Services Research—CINTESIS@RISE, 4200-450 Porto, Portugal

**Keywords:** presenteeism, non-academic university staff, COVID-19, higher education

## Abstract

Presenteeism negatively affects worker performance. We aimed to know the prevalence of presenteeism in non-academic university staff, identify health problems and associated factors, as well as explore the reasons that led to presenteeism during the COVID-19 pandemic. A cross-sectional study was conducted with a convenience sample of 332 non-academic staff. The Portuguese version of the Stanford Presenteeism Scale (SPS-6) was used, and socio-demographic and occupational data were collected. Participants were divided into groups according to the presenteeism cut-off score (no presenteeists, presenteeists with high job performance, presenteeists with low job performance). Multinomial regression was used to identify occupational and demographic characteristics associated with presenteeism. An open question replies analysis made it possible to explore the reasons for going to work while sick. Presenteeism was experienced by 30.1%. Presenteeism with high job performance was not associated with socio-demographic and work factors. Professionals who performed only physical work (OR = 9.4; 95% CI: 1.7; 51.0) and those who conducted hybrid work (OR = 4.1; 95% CI: 1.8; 9.6) showed a higher risk of belonging to the presenteeist group with low job performance. Financial reasons led professionals to work while sick. This study raises the importance of evaluating presenteeism in non-academic staff to create conditions for them to maintain high performance despite presenteeism and to intervene when there is low performance due to presenteeism.

## 1. Introduction

Apart from the loss of productivity and other costs associated with absenteeism (normally defined as being absent from work due to illness), which has been widely studied, nowadays, organizations are more and more concerned with the costs associated with presenteeism, defined as going to work in spite of being sick [1]. Previously, organizational policies focused on the minimization of absenteeism, and though presenteeism was seen as harmful, it was considered preferential because it represented a welcome act of organizational citizenship because when workers are physically present they still achieve some productivity when compared to absenteeists [1]. Health issues that lead to presenteeism are generally considered fairly mild or episodic because otherwise, people would be forced to stay at home [2]. Though it seems that these illnesses originate from low direct costs, in reality, indirect costs, which are barely perceptible to employers, must be considered. The literature tells us that presenteeism is approximately twice as expensive as absenteeism [3]. If, on the one hand, it can lead to a steep lowering of production due to the prevalence of health problems that can occur for years, on the other hand, over time, it becomes a risk factor, leading to the aggravation of the illness and, even, absence from work (absenteeism) [4]. For these reasons, there has been an increased interest in the study of presenteeism, which is seen as a global phenomenon [5].

Health issues and functional limitations are the main factors in the decision to go to work or not while ill [6,7,8]. It is well-known, for example, that there are workers who, in spite of being ill, consider themselves more resistant and stronger and who give less importance to the situation. They assume there is no danger involved and thus increase presenteeism [4,7].

However, other reasons may lead to this decision, such as financial difficulties, considering that he/she is irreplaceable, the desire not to overload colleagues with work, and feeling that the atmosphere at work sanctions absenteeism [3,4]. In a previous study, presenteeism was found to be a purposeful and adaptive behavior given the need to harmonize work demands and health limitations [6]. 

Past findings also suggested that socio-demographic and occupational characteristics might be associated with presenteeism, for example, sex/gender [3,9] and age [9], while others suggest no association or that the evidence was inconsistent [10,11,12,13]. In the context of university staff work, for example, the incidence of back pain in women was higher than that in men [14]. Regarding occupational characteristics, evidence was found of an association with presenteeism [11,15]. One of the reasons to work while sick is the achievement of organizational goals [4]. Added to this, the COVID-19 pandemic that is affecting the world population could be an even more accentuated problem for presenteeism [15] because, first, several risk factors, associated with presenteeism [1,3,15] may have worsened, such as, history of illness, concerns about financial and job insecurity [3,16]; second, organizations, having to maintain productivity, exert greater pressure on working hours and increase stress; it is also necessary to replace colleagues who are debilitated or sick, which leads to an aggravation of the risk of presenteeism, and of future problems of disease [16,17]; third, the pandemic leading in some cases to organizational adjustments which led to a reconfiguration of the workplace [18,19], such as telework (e.g., working outside the organization) [19,20] and increase in level of demand regarding self-management, which in turn could lead to the risk of presenteeism, and; finally, as diseases are circulating in organizations, presenteeism can be a risk factor contributing to the transmission of infectious diseases among professionals and to the pandemic situation [11]. Thus, organizations must strive for a culture of absence due to illness and minimize presenteeism [11]. 

There is a growing body of literature that recognizes the significance of presenteeism as a problem of public health. However, much of the research so far has focused on healthcare professionals on the front line, those most exposed to risk and health problems, with the most recent example being the pandemic [21,22]. A systematic review indicates the need to amplify studies in related organizational contexts [11]. For instance, the higher education sector has been the focus of great growth in recent decades, and university management is becoming increasingly competitive [23]. To compete successfully, universities need constantly changing and technically demanding work environments, which translates into a more qualified and highly skilled workforce [24]. The contribution of non-academic staff in supporting services is part of the quality of services provided by the Institution. Often, the implementation of new policies falls on these professionals, who are the first to feel the challenges institutions faces that translate into limited financial resources, an increasing number of students, new legislation and regulations, increasing bureaucracy, new technologies, and responsibilities. In addition, they play an important role in the university experience of students, as they are the first professionals with whom they deal directly, who contribute to their integration into the institution and provide them with assistance at different levels [25]. Adaptation to the COVID-19 pandemic has also brought transformations to the academic context [26]. A considerable number of university professionals (academic and non-academic) present a high level of exhaustion and work while ill for at least eight days. These circumstances do not benefit well-being [27]. However, if the staff indicated a satisfactory level of well-being, both the institution and students would benefit from this favorable context in terms of the support they would be offered. Typically, the focus of higher education research is academic staff and students rather than the support staff that work in academia. Given the significant changes in the profession, as described above, non-academic staff are potentially negatively affected by the work context. Valuing non-academic staff is crucial for the mission of universities, and an integrated culture of well-being must be promoted for all who work and study at the university [23]. The demands on non-academic staff are increasing to reflect the quality of services provided by institutions, but they can also lead to greater presenteeism. Up to now, far too little attention has been paid to presenteeism among these professionals. We have found one study that investigated presenteeism among non-academic staff. Family commitments and the unavailability of time to achieve specific goals were reported as important determinants of presenteeism among non-academics [25]. Therefore, we are convinced that it is relevant to expand knowledge about presenteeism in this population in order to envision a diagnosis and a possible intervention.

This study aimed to determine the prevalence of presenteeism in non-academic university staff, identify health problems and associated factors, as well as explore the reasons that led to presenteeism during the COVID-19 pandemic. Based on the theoretical evidence described above, we hypothesized, first, that non-academic staff are at high risk of presenteeism and, second, that compared with non-presenteeism workers, those with decreased performance due to presenteeism are affected by demographic and occupational characteristics.

## 2. Methods

### 2.1. Study Design

A cross-sectional observational study was conducted among a sample of Portuguese non-academic university staff by collecting data through an online questionnaire.

### 2.2. Subjects

The subjects were 322 non-academic staff from a Portuguese public university who voluntarily participated in the study between April and June of 2021.

### 2.3. Measures

Demographic (sex, age, academic qualifications, marital status, and household) and occupational characteristics (professional category, supervisor (yes/no), labor contract, years of work at institution, type of work, and place of work (previous month)) were collected. 

Presenteeism was evaluated from the Portuguese version [28] of the Presenteeism Scale (Stanford Presenteeism Scale—SPS-6) [29], which evaluated losses in work productivity due to health problems via two distinct domains: completed work (CW), which is the amount of work conducted under the effects and the causes of presenteeism, and avoided distraction (AD), which is the amount of concentration needed to work effectively while sick. They were both assessed via three items with five responses on a Likert scale from options “totally disagree” to “totally agree” [28,29]. In CW, the score 5, “totally disagree”, corresponds to the most unfavorable condition, and in the AD domain, it corresponds to the score 1, “totally agree”. For the latter, each numerical value of response was converted into the opposite value. 

The total score on SPS-6, which can vary from 6 to 30, is obtained by the sum of all the responses to all items of the two domains. Lower scores (from 6 to 18) denote reduced performance in work activities due to presenteeism; higher scores (from 19 to 30) denote better performance despite presenteeism [30]. The literature confirms good psychometric properties of this scale, including for the Portuguese population, the version we used in this study [28]. The results showed good internal consistency with a Cronbach’s α coefficient for the scale of 0.821 and for the subscales AD and CW of 0.774 and 0.832, respectively.

### 2.4. Statistical Analyses

The internal consistency of the scales was validated with Cronbach’s α coefficient. 

To compare the groups that displayed presenteeism with the group which did not display presenteeism, participants were divided according to presenteeism cut-off score: no presenteeism (the group that reported no health problem in the previous month and not having missed work) vs. lower presenteeism score (SPS-6 cut-off score ≤ 18) vs. higher presenteeism score (SPS-6 cut-off score > 18). This will allow institutions to better identify the group of people with lower performance due to presenteeism and to intervene as a priority. Participants’ characteristics between presenteeism groups were summarized with mean and standard deviation (SD) and absolute and relative frequency, as appropriate. Differences between groups were assessed using ANOVA and Pearson’s chi-square test or Fisher’s Exact test. Multinomial logistic regression was used in order to investigate the association between occupational characteristics and demographic presenteeism. The “no presenteeism” group was used as a reference category. All variables with *p* value < 0.2 at univariate analysis were included in the multivariable model. A level of 5% was considered significant. Furthermore, to confirm the direction of the odds ratios, a sensitivity analysis was performed by bootstrapping with 2000 replication.

In the above-mentioned survey, there is also an open question for professionals to indicate the main motive for going to work in spite of feeling ill. The treatment of the collected data was conducted using the method of content analysis proposed by Bardin [31]: organization of the analysis, exploration of the collected material (analysis of the text in regards to categories), treatment, and interpretation of results [31]. For the analysis of the individual replies, a grid was created (with a view to presenting data in an objective, organized and simplified manner) where we defined categories and subcategories, frequency of replies and their respective percentage, as well as the registration units followed with the letter P (participant) and the coding number assigned to the participant [31].

## 3. Results

### 3.1. Participants’ Characteristics

In Table 1 are described the participants’ characteristics. The mean age was 46 (SD 9), and 75.5% were female. Regarding academic qualifications, 68.9% had higher education, and most were married or were in an unmarried partnership (64.3%). Household consists on average of 2.8 people (SD 1.1). Regarding participants’ occupational characteristics, the group included 26 (8.1%) Operational Assistants, 89 (27.6%) Technical Assistants, 195 (60.6%) Senior Technicians, and 6 (1.9%) Informatics or Other. Most participants had a public contract 173 (53.7%), were not supervisors, 264 (82.0%), and the work type was mainly mental (61.8%). In the previous month, 105 (32.6%) mostly teleworked (at home), 79 (24.5%) worked at home and at the institution, and 138 (42.9%) worked mostly at the institution. The average length of employment at the institution was about 15 years. Presenteeism was experienced by 97 (30.1%) workers. For 54 (55.7%) of these workers, the global score on SPS-6 was equal to or lower than 18, that is, with an impaired performance at work due to presenteeism. Table 1 shows that groups did not differ in sex, age, academic qualifications, marital status, household, professional category, being a supervisor, labor contract, and years at the institution. 

### 3.2. Factors Associated with Presenteeism

Academic qualifications, type, and place of work (that reached the significance level of *p* < 0.2 at univariate analysis) were included in a multivariable model to identify independent factors associated with presenteeism. The results of the multivariate model are displayed in Table 2. In the multivariable model, type and place of work remained significantly associated with presenteeism. Professionals working both at the Institution and at home (OR = 4.1; 95% CI: 1.8; 9.6) were more likely to have reduced performance of work activities due to presenteeism (Presenteeism ≤ 18) compared with those that worked mostly or always in telework. Those whose work was only physical (OR = 9.4; 95% CI: 1.7; 51.0) were also more likely to have reduced performance of work activities due to presenteeism (Presenteeism ≤ 18) compared with workers in a mental and physical job.

### 3.3. Health Problems

**Figure 1:**
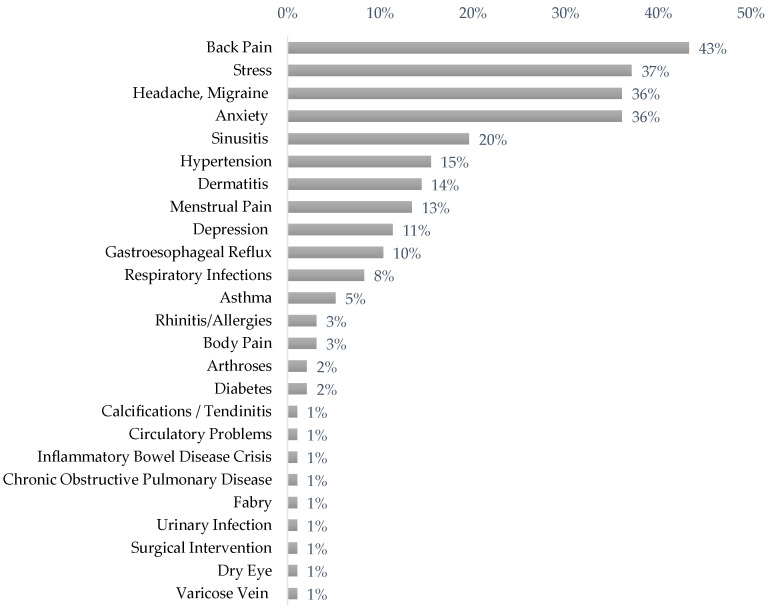
Prevalence of common health problems reported by presenteeist participants.

The most common health problems reported by participants at work in spite of illness were back pain (42.3%), stress (37.1%), headache, and anxiety (36.1%) (Figure 1).

### 3.4. Reasons for Presenteeism

**Figure 2:**
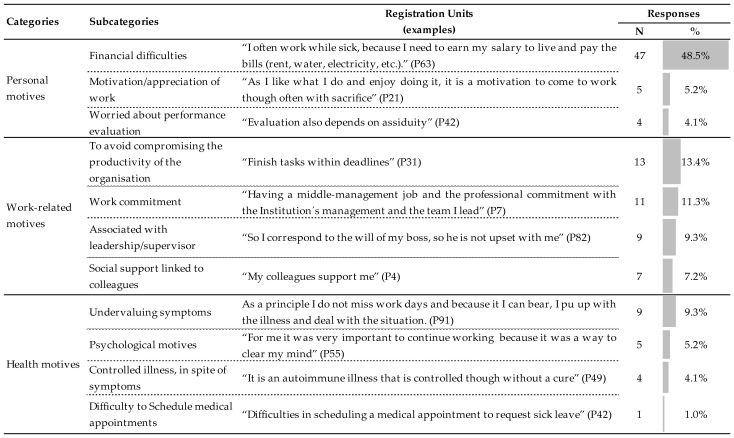
Categories, subcategories, registration units, and frequency of the Reasons for Presenteeism.

From the participants who stated they attended work while ill (*n* = 97), we obtained a total of 115 answers. We identified three broad categories that support the reasons associated with presenteeism: personal, work, and health, as well as 11 subcategories. “Financial difficulty” was the most frequent reason (48.5%) for going to work ill. The subcategories “to avoid compromising the productivity of the organization” (13.4%) and “work commitment” (11.3%) also stand out. The positive reasons associated with presenteeism, such as “motivation/appreciation of work” (5.2%) or psychological motives (5.2%), should also be mentioned (Figure 2).

## 4. Discussion

Being present at work in spite of being ill implies a future risk, not only because it may make it necessary to be absent due to illness for longer periods, but it may also lead to undervaluation of illness [32]. Thus, it is necessary to understand, recognize, and prevent this behavior. This study provided new information about the global phenomenon of presenteeism, confirming its existence among non-academic staff. It also provided further evidence of the effect of demographic and occupational characteristics on presenteeism in these professionals. 

This study showed a prevalence of presenteeism of 30.1%, which confirms that it is a problem that may affect various professional groups, including public university non-academic staff. Moreover, 55.7% of the professionals who reported working while sick were included in the low presenteeism group, i.e., who reported decreased performance at work due to presenteeism. This demonstrates the importance of assessing presenteeism in a university context but also of identifying and monitoring high- and low-performance presenteeism groups, given the risk of the latter condition developing into more serious situations for both professionals and organizations. The management of presenteeism (highlighting the health of the professionals) may provide organizations with a competitive advantage by investing in measures to protect their valuable human resources [2]. 

One review expressed concern about presenteeism, showing rates ranging from 30% to more than 90% [33]. These results mean that professionals work while feeling sick and, therefore, with health conditions that may worsen over time [32], which can lead to high costs for organizations and professionals. In our study, among the main physical symptoms of presenteeism, we highlight back pain and headaches; among the main psychological symptoms, we highlight anxiety and stress. Other problems previously identified in other studies and in other areas of activity which led to presenteeism were rheumatoid arthritis, insomnia conditions, and back pain [34]. Another study carried out among university professionals point to the prevalence of back pain; Their more sedentary work exposed them to occupational risks, such as long hours of sitting, which is related to back pain that affects their well-being [14]. The authors also add that due to this situation, they are more prone to depression [14]. Additionally, stress at the university workplace and in other areas of activity has negative consequences for the well-being of academic staff, their families, the organization, and their work colleagues [35]. Some of these problems may originate from the work context itself. There is evidence that stress and sleep, for example, are among the most common factors (alone or together) associated with primary headaches [36]. Professionals experience stress when there are strong work demands compared to their personal capacity to cope with them. Due to prolonged stress, workers may develop severe mental and physical health problems (e.g., musculoskeletal disorders and cardiovascular diseases) [37]. In turn, professionals with mental health problems are expected to present a high prevalence of presenteeism due to the fear of being stigmatized in the work context [4]. 

Identifying factors associated with presenteeism, especially those related to work, may have a dual benefit in reducing presence at work while sick but also in absence. This study also assessed the socio-demographic and work-related factors between the high and low presenteeism groups (higher and lower performance perception) and the group of professionals who did not report presenteeism. This may help to better identify factors associated with presenteeism and specifically with low and high presenteeism groups. Interestingly, none of these factors were associated with the highest-performing group despite presenteeism when compared to the group without presenteeism. This means that socio-demographic and work factors in high presenteeism do not increase the perception of lower performance even when working while ill. In this respect, there is recent evidence, in the context of the COVID-19 pandemic, that, for example, Swedish self-employed workers did not show a significant relationship between sickness presenteeism and age, gender, and education [13]. Conditions should be created to allow professionals to maintain high performance even when they are sick. On the other hand, we see that sex is the socio-demographic characteristic that the literature draws attention to [3,14,38]. According to a review of the literature, one reason could be associated with the traditional role of women, which still exists in various societies. Apart from paid employment, [women in these societies] also do housework, take care of the children, and have little time to care for themselves [14,39].

In turn, socio-demographic and work-related factors were associated with presenteeism with low performance. The type and place of work were identified as factors that may influence the perception of performance due to presenteeism. Specifically, professionals with only physical jobs are more likely to belong to the low presenteeism group than those with both physical and mental work. This demonstrates that physical work can be associated with greater presenteeism [40]. In our study, physical work is more evident in the occupational categories of operatives and assistants. Similarly, professionals who perform hybrid work, that is, who work remotely and also go into the workplace, compared to those who perform only telework, presented a higher risk of belonging to the group of low performance due to presenteeism. This new work format, the hybrid model, can be perceived as being poorly structured and ambiguous if compared to full-time face-to-face or remote work [41]. This might explain our participants’ sudden change of conditions due to COVID. There were inevitable necessary adaptations to a new context. The literature indicates that academic professionals recounted stressful situations and some lack of clarity at the organizational level [26]. A study conducted with professionals from three Swedish public institutions reported that there are challenges regarding risks of contagion from COVID-19, which put pressure on organizations to ensure a better physical environment. Distancing between professionals is needed increasing safety at the workplace, as well as organizations are expected to assist professionals with guidelines and means to meet the needs for appropriate ergonomics of the home office. Other challenges included the decision concerning the days when professionals go to work to carry out the tasks adapted to face-to-face or remote work [41]. In another study conducted during the COVID-19 pandemic with students and professionals (teaching and staff) at a Finnish university, staff members reported being excited to have learned new working practices related to remote work. However, this also led to pressures and exhaustion [42]. The hybrid model could be seen as a favorable format for higher education professionals. Interestingly, a study conducted during the COVID-19 quarantine with Lithuanian professionals in the public sector, which includes school and higher education teachers, points out that doing telework two days a week may be synonymous with motivation, and may not be detrimental to the quality of relationship and work produced by the professional which does not occur with those who work remotely most of the time [43]. If, on the one hand, working in person in the organization is favorable because there is a better ergonomic configuration of the workplace, face-to-face socialization with colleagues, and natural physical activity due to various types of travel (e.g., to and from the organization) [41], on the other hand, according to a review, working at home means being able to reconcile professional and personal life, work flexibility, lower health risks (e.g., because of contagion), and saving travel time [44]. Thus, hybrid working, a model with a future, makes it possible to balance the advantages of working at home and in the organization and consequently increase productivity [45]. However, as it is more difficult to identify, organizations should be aware of the probable risks of this type of work (home-based telework) [20]. 

It is important to bear in mind other potential variables that may demonstrate differences between the groups belonging to our study. Another study in a university context suggests the need to adopt procedures to avoid psychosocial risks among university professionals and not only consider the well-being of students [23]. According to evidence, organizations must privilege a culture of absence due to illness and minimize presenteeism [11]; equally relevant is occupational health, focusing on the promotion of better work contexts that protect the health of professionals [46]. Social support in the workplace, including that of supervisors and co-workers, may help to prevent presenteeism as it favors interpersonal relations, a positive environment in the workplace, and the possibility of flexible timetables, where this is possible [47], and having time for self-care, avoiding going to work when ill (because a member of the group may be temporarily substituted by reorganization of the team) [3]. 

The results of the qualitative analysis allowed us to identify the underlying reasons why non-academic university staff work even if they are ill. Personal, work, and health-related motivations were the categories identified in this study. Experiencing financial difficulties was the most frequently mentioned reason. Professionals cannot afford to have their salaries reduced, and this situation overlaps with health problems [48]. Other more prevalent reasons were to avoid compromising the organization’s productivity and work commitment. Organizations that are short of human resources may need to extend not only their time but also their workload. This might have been reinforced during the aforementioned COVID-19 pandemic due to colleagues being sick. Thus, pressure and stress may lead to a higher risk of presenteeism [17]. It was interesting to note that professionals may present therapeutic presenteeism, as it can be positive for self-esteem to have a favorable work environment, to enjoy the social support of the team, and create a reputation as a conscientious professional with a sense of responsibility (however not very productive). For example, when professionals are aware of a consistent threat to their own health, they may demonstrate presenteeism if they consider that the negative effects of it may be less than the positive consequences [6,7]. Some of the statements found in our study indicating a preference for working were: “For me it was very important to continue working, because it was a way to clear my mind.”; “My colleagues support me.” Presenteeism can be a sustainable option to ensure performance, even under adverse health conditions, if the workplace is favorable and there is flexibility in terms of labor resources that enable adaptation. In this way, there is a necessary balance between the demands of professional performance and health limitations (adaptive presenteeism) [6]. On the other hand, work motivation can also lead to presenteeism, for example, when the professional is involved in work and its performance demands while feeling ill, but without increasing the burden on his/her health (functional presenteeism) [6]. Both work demands and personal and work resources lead to presenteeism; this is due to the existence of health problems and motivation, with positive attitudes being synonymous with commitment, satisfaction, and engagement [4].

## 5. Limitations and Future Research

With opportunities for future research, our study has some limitations. First, the participants in our study are limited to a single public university. Hence, it is not clear whether the results are general among other higher education institutions, even though it includes the participation of big universities. Additionally, being carried out during the period of the COVID-19 pandemic constitutes a comparative framework for future investigations. Second, the cross-sectional design and the convenience sample do not allow us to draw conclusions concerning the causal relationship between factors. Third, our results on presenteeism are based on a self-perception measure. Nevertheless, SPS-6 was applied previously, and it is a reliable and widely used scale. Finally, even though a wide confidence interval was noted in the final analysis due to the low numbers of participants exposed to physical work, the direction of the ORs was also confirmed through bootstrap analysis.

While we believe our results provide the starting point for any discussion of non-academic staff, presenteeism also raises a number of other important questions to be addressed in further research. In future research, it is important to expand the sample to other universities, and further relations can be tested by integrating other domains such as university size and type of professional career. On the other hand, any analysis of presenteeism must consider other potential factors that influence the phenomenon and can better characterize professionals with high and low presenteeism. This development will help higher education institutions to minimize presenteeism and to introduce new opportunities for the non-academic university staff. In addition, prospective studies are needed to evaluate possible causes of presenteeism.

## 6. Conclusions

The existence of presenteeism in non-academic staff in public universities, as well as the presence of low and high-performance groups due to presenteeism is a reality. The comparison between groups allowed us to demonstrate the similarity of the high-performing presenteeism group with the group without health problems in the demographic and work factors considered. However, the same is not true in the low performance due to presenteeism (condition). Performing only physical tasks and hybrid work may increase perceptions of lower performance due to presenteeism. It is necessary for public university institutions to rethink the working conditions that should be adjusted to the needs of professionals, especially those needs which emerged in a pandemic context, such as face-to-face and hybrid work, where social support at work can minimize health problems and attenuate the reasons that lead professionals to work even if they feel ill, avoiding or alleviating presenteeism.

Going to work while ill depends mainly on financial difficulties, responsibility, and devaluation of symptoms. Curiously, this study demonstrates the relevance of considering the positive effects of presenteeism, such as helping to forget and overcome problems—until now, the focus has mainly been on its negative effects and how to reduce the behavior. 

These findings raise the importance of evaluating presenteeism in non-academic staff to create conditions to maintain high performance despite presenteeism and intervene in low performance due to presenteeism.

## Figures and Tables

**Table 1:** Participants’ Characteristics by Groups of Presenteeism.

Characteristics	Presenteeism				Total
	No (N = 225)	Higher Score (N = 43)	Lower Score (N = 54)	*p* Value	(N = 322)
Sex, N (%)				0.362	
Female	174 (77.3)	29 (67.4)	40 (74.1)		243 (75.5)
Male	51 (22.7)	14 (32.6)	14 (25.9)		79 (24.5)
Age, Mean (SD)	45.9 (9.3)	45.4 (9.2)	46.7 (7.7)	0.748	46.0 (9.0)
Academic Qualifications N (%)				0.087	
≤3rd Cycle	12 (5.3)	7 (16.3)	5 (9.3)		24 (7.5)
Secondary	50 (22.2)	11 (25.6)	15 (27.8)		76 (23.6)
Higher Education	163 (72.4)	25 (58.1)	34 (63.0)		222 (68.9)
Marital Status, N (%)				0.788	
Divorced, Separated, Widow/er, or Single	83 (36.9)	14 (32.6)	18 (33.3)		115 (35.7)
Married or unmarried partnership	142 (63.1)	29 (67.4)	36 (66.7)		207 (64.3)
Household, Mean (SD)	2.9 (1.2)	2.8 (1.2)	2.7 (1.1)	0.688	2.8 (1.2)
Occupational Category, N (%)				0.240	
Operational Assistant	13 (5.8)	6 (14.0)	7 (13)		26 (8.1)
Technical Assistant	61 (27.1)	13 (30.2)	15 (27.8)		89 (27.6)
Informatics	6 (2.7)	0 (0.0)	0 (0.0)		6 (1.9)
Senior Technician	142 (63.1)	23 (53.5)	30 (55.6)		195 (60.6)
Other	3 (1.3)	1 (2.3)	2 (3.7)		6 (1.9)
Supervisor, N (%)				0.694	
No	182 (80.9)	37 (86.0)	45 (83.3)		264 (82.0)
Yes	43 (19.1)	6 (14.0)	9 (16.7)		58 (18.0)
Labor Contract, N (%)				0.299	
Public	118 (52.4)	21 (48.8)	34 (63.0)		173 (53.7)
Private	107 (47.6)	22 (51.2)	20 (37.0)		149 (46.3)
Years of work at the institution, Mean (SD)	15.3 (10.0)	14.8 (10.3)	16.1 (7.2)	0.800	15.4 (9.6)
Type of Work, N (%)				0.001	
Mostly physical	3 (1.3)	2 (4.7)	5 (9.3)		10 (3.1)
Mostly mental	146 (64.9)	18 (41.9)	35 (64.8)		199 (61.8)
Physical and mental	76 (33.8)	23 (53.5)	14 (25.9)		113 (35.1)
Place of Work (previous month), N (%)				0.002	
Mostly or always at home (telework)	87 (38.7)	8 (18.6)	10 (18.5)		105 (32.6)
Same time at home as at Institution	48 (21.3)	10 (23.3)	21 (38.9)		79 (24.5)
Mostly or always at Institution	90 (40.0)	25 (58.1)	23 (42.6)		138 (42.9)

**Table 2:** Multinomial logistic regression analysis of socio-demographic and work factors associated with presenteeism.

No Presenteeism (Reference Category)	Presenteeism > 18		Presenteeism ≤ 18	
Characteristics	OR (95% CI)	*p* Value	OR (95% CI)	*p* Value
Academic Qualifications				
Up to 3rd cycle	2.2 (0.7; 7.2)	0.183	1.4 (0.3; 5.8)	0.672
Up to Secondary	1.0 (0.4; 2.3)	0.960	1.3 (0.6; 2.7)	0.549
Higher Education	1 (Ref.)		1 (Ref.)	
Place of Work (previous month)				
Mostly or always at home (telework)	1 (Ref.)		1 (Ref.)	
Same time at home as at Institution	2.0 (0.7; 5.6)	0.171	4.1 (1.8; 9.6)	0.001
Mostly or always at Institution	2.3 (0.9; 5.7)	0.070	2.1 (0.9; 5.0)	0.081
Work Type				
Physical and Mental	1 (Ref.)			
Physical	1.4 (0.2; 10.1)	0.723	9.4 (1.7; 51.0)	0.009
Mental	0.6 (0.3; 1.2)	0.161	1.7 (0.8; 3.7)	0.190

Note: Reference category for dependent variables is the group that reported not having any health problems in the previous month and who were present at work.

## Data Availability

The data are not publicly available due to privacy issues. Requests to the datasets should be directed to Sónia Magalhães, soniamagalhaes73@gmail.com.
